# Klippel–Trenaunay Syndrome: Employment of a New Endovascular Treatment Technique—Mechanochemical Ablation Using the Flebogrif System

**DOI:** 10.3390/jcm11185255

**Published:** 2022-09-06

**Authors:** Piotr Terlecki, Karol Terlecki, Stanisław Przywara, Marek Iłżecki, Michał Toborek, Radosław Pietura, Paweł Maga, Mikołaj Maga, Tomasz Zubilewicz

**Affiliations:** 1 Department of Vascular Surgery and Angiology, Medical University of Lublin, 20-081 Lublin, Poland; 2 Department of Radiography, Medical University of Lublin, 20-081 Lublin, Poland; 3 Angiology Department, Medical Faculty, Jagiellonian University Medical College, 30-688 Krakow, Poland; 4 Clinical Department of Angiology, University Hospital in Krakow, 30-688 Krakow, Poland

**Keywords:** Klippel–Trenaunay syndrome, mechanical and chemical ablation, Flebogrif, marginal vein

## Abstract

Background: Klippel–Trenaunay syndrome (KTS) is characterized by a triad of symptoms; varicose veins and venous malformations (VMs), capillary malformations (port-wine stain), and soft tissue and bone hypertrophy. Herein, we retrospectively studied six patients with KTS who underwent treatment with the Flebogrif system and evaluated their outcomes. Methods: Six KTS patients aged 16–22 years who had undergone 18 non-thermal ablations using the Flebogrif system were enrolled. All patients underwent multistage foam sclerotherapy with 3% polidocanol at 3–4-week intervals. Results: Venous clinical severity score (VCSS) analysis showed improvement in the patients’ clinical condition. All patients reported a significant improvement in aesthetic outcomes. One patient presented with recanalization of ablated marginal veins during the 24-month follow-up period. Patients could return to full activity within 7–10 days after the procedure. None of the patients experienced serious systemic complications. Conclusion: The use of the Flebogrif system in treating various forms of chronic venous insufficiency, including in patients with KTS, provides a high success rate with a high closure rate.

## 1. Introduction

According to the 2018 International Society for the Study of Vascular Anomalies, Klippel–Trenaunay syndrome (KTS) is a vascular malformation associated with other anomalies [1]. It is characterized by a triad of symptoms, including varicose veins, venous malformations (VMs), capillary malformations (port-wine stains), and soft tissue and bone hypertrophy. Recent studies have linked the etiology of KTS to somatic mutations in the phosphatidylinositol-4–5-bisphosphate 3 kinase, catalytic subunit (PIK3CA) gene. This leads to the activation of phosphatidylinositol-3-kinase (PI3K)/protein kinase and cell overgrowth by dysregulation of the mTORC2 pathway [2,3]. Mutations occur in the embryological stage of development involving angiogenesis, reflecting findings seen in this condition. KTS is now grouped under the umbrella of similar overgrowth syndromes—PIK3CA-related overgrowth spectrum (PROS). Several overgrowth syndromes with overlapping clinical manifestations involving a number of mutations in the PIK3CA gene have been identified. In rare instances, translocations of chromosomes 5–11 and 8–14 have been reported [4].

Its incidence is estimated to be 2–5 out of 100,000 cases, and it is more common in men than in women (1.5:1). A diagnosis is established if patients present with at least two of the triad symptoms [5].

Due to poor surgical outcomes, asymptomatic patients are limited to compression treatment. Analgesics, antibiotics, and low-molecular-weight heparins (LMWHs) are commonly used to treat complications, such as cellulitis or thrombophlebitis [6].

Sclerotherapy using polidocanol (POL), sodium tetradecyl sulfate (STS), microfoams, or absolute alcohol can be used as a supplementary treatment for varicose veins and VMs; however, these are less effective in treating major lesions, including trunk insufficiency [7,8].

The role of thermal and non-thermal endovenous ablation, which has widely recognized efficacy in treating lower extremity varicose veins, has not been commonly used in KTS patients due to its low frequency. However, in the treatment of varicose veins, endovenous ablation is preferred over surgical procedures, which are limited by lower efficacy [9,10,11].

These methods have certain limitations, including the maximum transverse dimension of the treated vein, which should not exceed 10 mm. The introduction and effective use of the Flebogrif system has enabled the therapy of veins with a markedly larger diameter, which is noted in most patients with KTS [12].

## 2. Materials and Methods

The patients qualified for interventional treatment based on Doppler examination and MRI venography. The diameters of the treated veins were measured in an upright position and oscillated as follows: marginal veins (7–16 mm; median 11 mm), GSVs (6–9 mm; median 8 mm), and SSVs (6–7 mm; median 6.5 mm).

**Figure 1:**
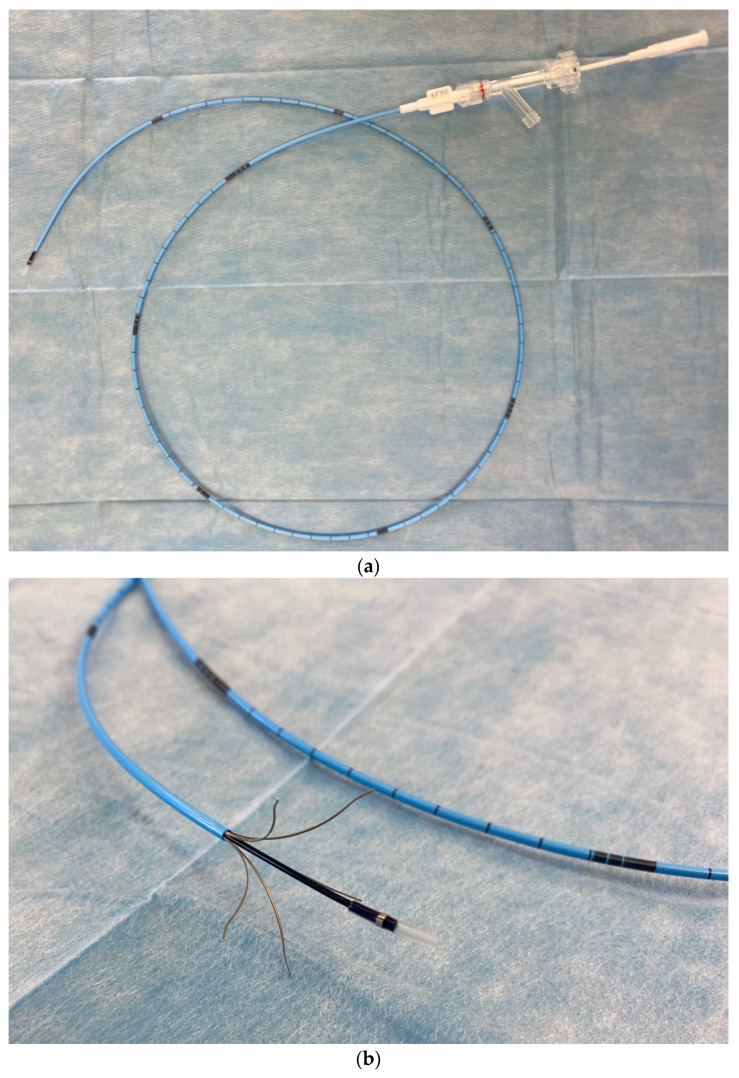
(**a**) Flebogrif catheter construction details—neutral phase. (**b**) Flebogrif catheter construction details—active phase.

The procedures were performed under local anesthesia at the site of vein puncture, with the introduction of a 6-Fr sheath using 1% lignocaine. Flebogrif^®^ is a system comprising two coaxial catheters (Figure 1a,b). The internal catheter is fitted with five cutting, self-expanding hooks at the top, which is the operating part of the device. The external system serves as the introduction system for the entire device. In the active phase, when the external catheter is moved towards the handpiece, it releases the operating part of Flebogrif. By steadily retracting the device concurrent with the administration of a sclerosing agent (polidocanol), the endothelium undergoes mechanical and chemical destruction. The immediate effect is the occlusion of the venous lumen. The vein becomes fibrotic and fully closes over several weeks. The device was placed under ultrasound (US) guidance 2 cm from the ostium of the treated vein to the deep vein system.

**Figure 2:**
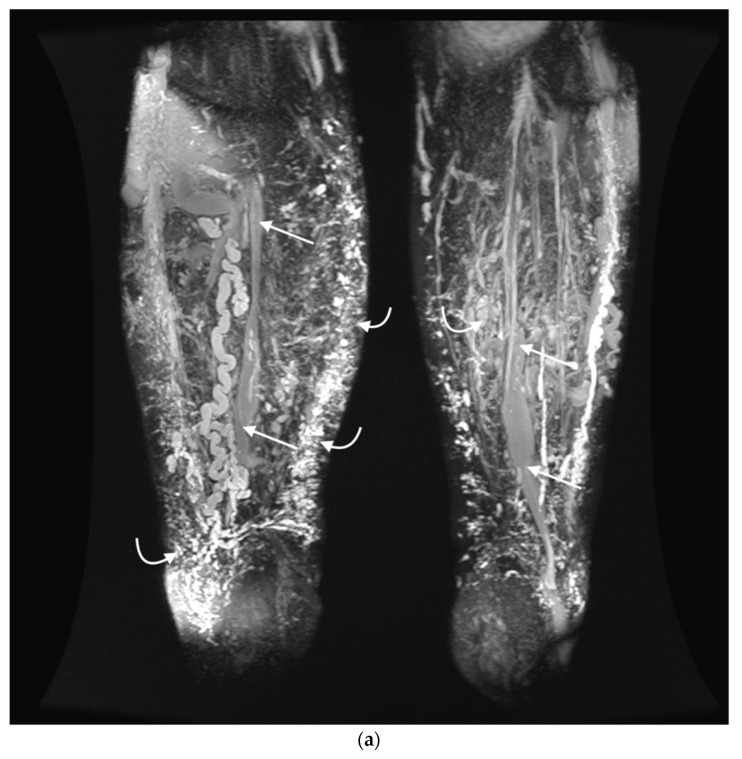

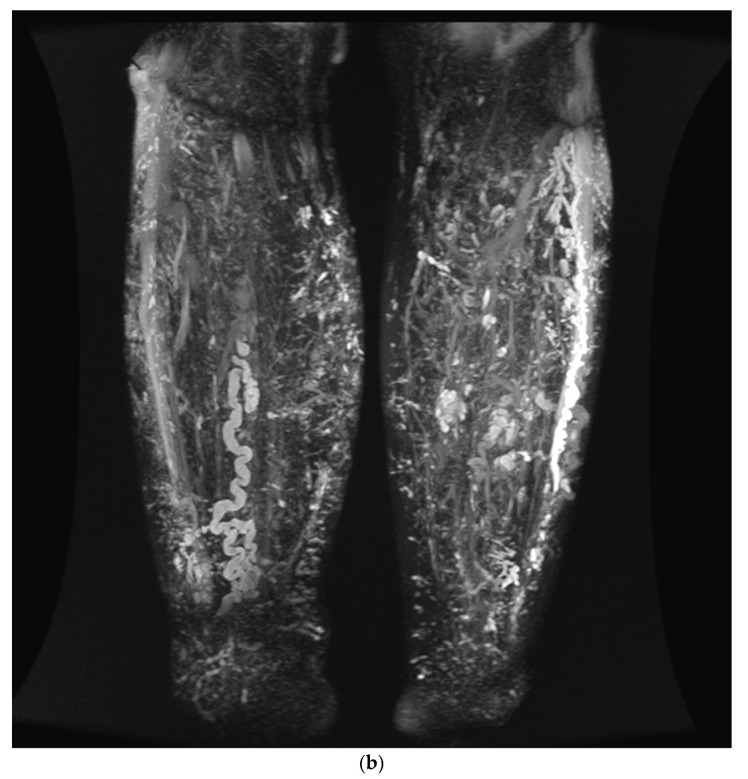
(**a**) MIP reconstruction from 3D T2-weighted FSE sequences (CUBE; GE Healthcare) in the coronal plane at the thigh before treatment. The occlusion of SSVs (straight arrows) with significantly fewer minor, abnormal veins in the subcutaneous tissue (bent arrows) after treatment is remarkable. MIP is an algorithm where voxels with the highest signal intensity overlap with subsequent scan slices. To provide optimal visualization of treatment outcomes, an MIP image was created using 1.6 mm thick slices, resulting in an image of vessels from an 84 mm thick area. (**b**) MIP reconstruction from 3D T2-weighted FSE sequences (CUBE; GE Healthcare) in the coronal plane at the thigh after treatment.

The system was released by sliding the external cover off from the internal pin. Once the five arms of the operating part were released with sharp hooks at their tips, the system was steadily pulled back peripherally, leading to endothelial scarification of the vein while simultaneously administering 3% POL foam (1 mL per 5 cm of the treated vein). Up to two superficial vein trunks were ablated simultaneously, which was complemented by foam sclerotherapy of varicose veins and VMs using up to 10 mL 3% POL foam per session. Due to the size of the varicose veins and VMs, the first sclerotherapy was performed with tumescence, thereby increasing the area of local effects of the sclerosant on the venous wall, as previously described by Cavezzi and Devereux [13,14]. Subsequent foam sclerotherapy sessions for varicose veins and VMs were performed at 4-week intervals. Grade 2 compression stockings were used for at least 10 days after the procedure. Due to the lack of additional risk factors for venous thromboembolism (VTE) during the perioperative period, standard anticoagulation prophylaxis such as LMWH was not employed. Retrospective follow-up was performed for 12–24 months (mean, 20 months) after treatment. Follow-up examinations were performed at 1, 3, 6, 12, and 24 months post-procedure. Technical success was defined as complete vein occlusion. After ablation with Flebogrif, the venous insufficiency index and venous clinical severity score (VCSS) [15] were evaluated (clinical success). Additionally, all patients underwent screening and follow-up MRI examination after 12 months to objectively evaluate treatment efficacy. The extensiveness of VMs was evaluated using T2-weighted sequences with adipose tissue saturation and thin-slice sequences with gradient-echo T2/T1 (Figure 2a,b). The study was approved by the Bioethics Committee of the Medical University.

## 3. Results

A retrospective analysis of six patients with Klippel–Trenaunay syndrome was performed. The patients were 16–22 years old (median, 19.5 years) and underwent a total of 18 non-thermal ablations with the Flebogrif system (Balton Company Ltd., Warsaw, Poland), including 11 in marginal veins, five great saphenous veins (GSV), and two small saphenous veins (SSV). All patients underwent multiple-stage foam sclerotherapy with 3% POL for varicose veins at 3–4-week intervals. This staged protocol was applied because of the limitation of the total volume of sclerosant that could be administered during a single procedure. The demographic information of the patients is presented in Table 1.

**Figure 3:**
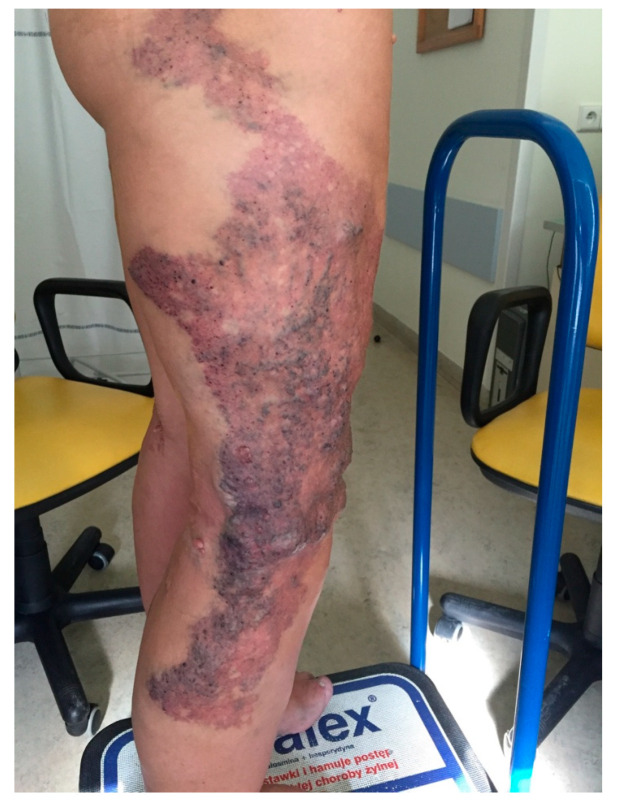
Clinical presentation pre-ablation with Flebogrif.

**Figure 4:**
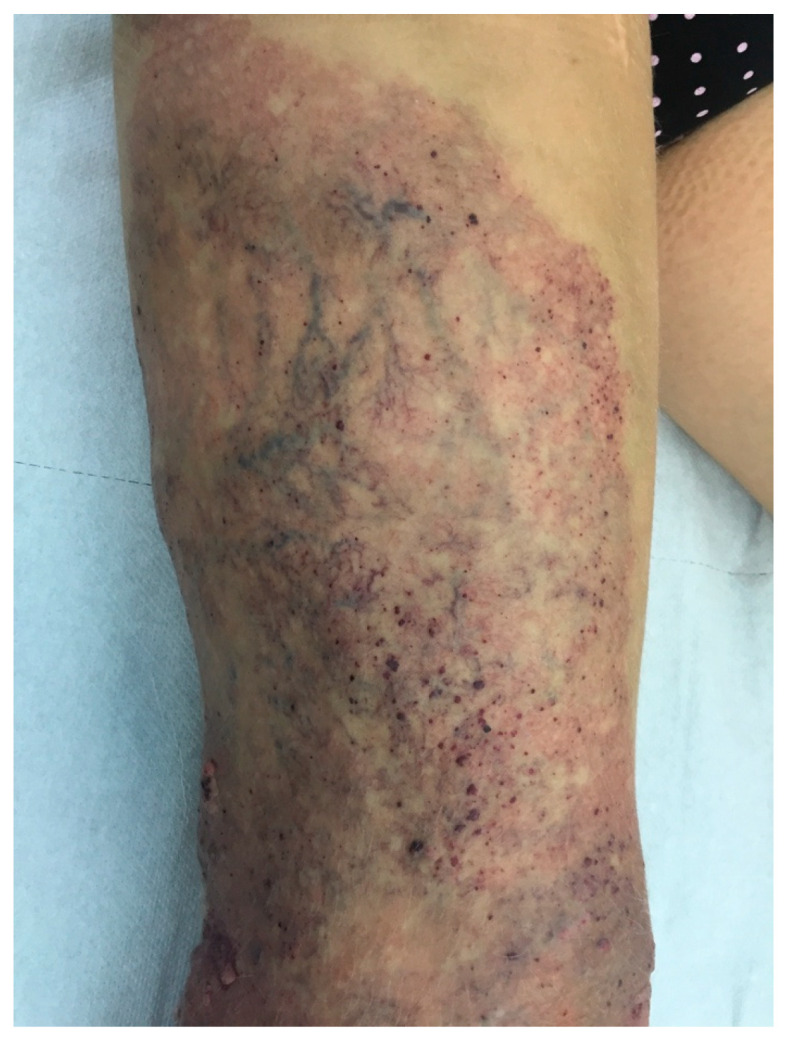
Final aesthetic outcome 12 months after ablation, followed by nine sessions of foam sclerotherapy.

Immediate technical success was achieved in all patients, with a procedural time of 7–16 min (mean, 11 min). The patients could walk immediately after the procedure and be discharged after an hour of outpatient observation. The patients required 5–9 supplementary foam sclerotherapy sessions of varicose veins and VMs, mostly due to their size in tumescence, to achieve better effects of the sclerosant on the vascular wall during the first session. The analysis of the results obtained using the VCSS demonstrated improvements in the patients’ clinical conditions, both between individual follow-up visits and from day 0 of evaluation. The intensity of the clinical symptoms remained stable from the 6-month visit onwards. The results of the 24-month follow-up period are shown in Table 2. The aesthetic effect also significantly improved in all patients, as shown in the photographic documentation (Figure 3 and Figure 4). In one female patient, recanalization of the ablated marginal veins was observed on MRI findings during the 24-month follow-up. The patient resumed full physical activity and returned to work 10 days after ablation. None of our patients suffered from serious systemic complications such as deep vein thrombosis or pulmonary embolism. Bruising or hematomas were observed in every patient postoperatively, but they resolved spontaneously, after a period of 10–14 days.

## 4. Discussion

The role of endovascular and minimally invasive interventions in the treatment of KTS has not been fully explored, owing to its low incidence. The Mayo Clinic team has reported the most extensive experience in this area. Gloviczki et al. reported that surgical treatment is absolutely indicated in patients with KTS if they experience recurring bleeding, trophic ulcers resistant to combination therapy (pharmacological and compression therapy), and VTE. The relative indications include pain, functional impairment, extremity edema, and cosmetic considerations. Thermal ablations, including laser and radiofrequency (RF), supplemented with sclerotherapy or malformation embolization using absolute alcohol, are currently recommended [16]. Endovascular treatment is considered safe and effective for various forms of chronic venous insufficiency and offers significant advantages over surgical methods, including rapid convalescence and the resumption of work duties.

Several reports have previously described the use of endovenous ablation in the treatment of embryonic veins in children with KTS. In 2008, Frasier et al. [17] described the use of endovenous RF ablation to treat GSV and marginal vein insufficiency in three patients with KTS. In one case, the ablation effect of venous closure was not achieved despite several attempts. In 2017, Patel et al. [18] reported laser ablation procedures in 35 children, including eight with KTS, with an 88% efficacy rate and only minor complications, such as transient pain in one patient. Very good results were also found in the Bittles study, which reported the cases of three children treated by laser with a 100% occlusion rate in 2–3 months of observation [19]. ClariVein, another mechanochemical ablation device, was successfully used in a 17-year-old boy with Cloves syndrome and an incompetent anomalous marginal vein extending from the dorsum of the left hand to the axilla [20].

Thermoablation techniques used to treat marginal veins remain debatable due to their anatomic superficial location, which is linked to a high risk of skin burns and discoloration [21]. Owing to the development of non-thermal venous ablation methods (Clarivein, Flebogrif, n-butyl cyanoacrylate) that are not affected by thermal injury, the treatment of superficial veins with good aesthetic effects has become possible [8,9].

Mechanical–chemical ablation is based on a combined mechanism of action. Depending on the treatment system, this technique can trigger vasospasm, enabling a deeper and more potent effect of the sclerosant, ultimately leading to venous fibrosis. Varicose vein treatment of the lower extremities using the Flebogrif catheter is a new mechanical–chemical ablation system characterized by a simple construction and a short learning curve during the 2-year follow-up [22].

An extremely important advantage of this system is the spacing of the cutting elements after they are released from the sheath. The spacing reaches a diameter of 28 mm, making it possible to apply in patients whose vein diameter exceeds 10 mm, which is often observed in patients with KTS.

Although all present patients required at least two stages of treatment with Flebogrif and several supplementary foam sclerotherapy sessions, the procedures went well. In addition, this method allows for the rapid resumption of full physical activity after each treatment stage.

## 5. Conclusions

Using Flebogrif to treat various forms of chronic venous insufficiency, including Klippel–Trenaunay syndrome, ensures a high occlusion index, which is an indicator of treatment efficacy. This simple system does not require a lengthy learning curve, and the procedure is well tolerated by patients because it is minimally invasive. This technique expands and complements the commonly used endovenous treatment systems; however, further studies and follow-up are necessary, particularly for patients with KTS.

## Figures and Tables

**Table 1:** Demographic data of the study participants.

Characteristics	No. (%) or Mean
Male sex	4 (66%)
Age (years)	16–22 (median 19.5)
CEAP clinical grade	
C3	1 (16%)
C4	3 (50%)
C5	1 (16%)
C6	1 (16%)
Clinical manifestation	
Varicose veins/venous malformations	6 (100%)
Port-wine stains	6 (100%)
Tissue/bone hypertrophy	6 (100%)
Treated vein	
Great saphenous vein (GSV)	5 (28%)
Small saphenous vein (SSV)	2 (11%)
Marginal vein (MV)	11 (61%)
Treated vein diameter (mm)	
Great saphenous vein (GSV)	6–9 (median 8)
Small saphenous vein (SSV)	6–7 (median 6.5)
Marginal vein (MV)	7–16 (median 11)
Procedure	
Mechano-chemical ablation (Flebogrif)	18
No. of sclerotherapies per patient	3–6 (mean 5)

**Table 2:** Follow-up data of the study participants.

	Day 0	1 Month	3 Months	6 Months	12 Months	24 Months
Any adverse events	0	0	0	0	0	0
Any recanalization	0	0	0	0	1 (partial)	0
No of Flebogrif ablations	6	5	1	0	0	0
No of sclerotherapy sessions	6	6	6	4	4	3
VCSS (mean)	9–16 (12)	6–12 (9.3)	5–11 (8)	5–9 (7)	5–10 (7)	5–9 (7)

## Data Availability

Not applicable.
